# Pyoderma Gangrenosum: A Case Highlighting the Importance of Timely Diagnosis

**DOI:** 10.7759/cureus.57762

**Published:** 2024-04-07

**Authors:** Rabia Rukhshan, Chioma Eliogu, Anuja Mistry, Simran Brar, Arshad Ali Mohd, Lela Ruck

**Affiliations:** 1 Internal medicine, Texas Tech University Health Sciences Center El Paso Paul L. Foster School of Medicine, El Paso, USA; 2 Internal Medicine, Texas Tech University Health Sciences Center El Paso Paul L. Foster School of Medicine, El Paso, USA; 3 General Medicine, Deccan College of Medical Sciences, Hyderabad, IND

**Keywords:** therapeutic delays, early identification and diagnosis, amputation, biopsy, pathergy, pyoderma gangrenosum

## Abstract

Pyoderma Gangrenosum (PG) is a distinctive dermatologic condition characterized by recurrent inflammatory ulcers, often manifesting with violaceous borders and undermined edges. We describe a 40-year-old male who presented with acute on chronic necrotic ulcer of the left index finger following foreign body penetration. Despite multiple emergency department visits and treatments for presumed recurrent cellulitis, including various debridements, his condition persisted without symptomatic relief. A high index of clinical suspicion, due to recurrent presentations and potential pathergy, prompted an excision biopsy which confirmed Pyoderma Gangrenosum (PG). Regrettably, due to delays in appropriate management, the patient chose amputation because of intolerable pain, highlighting the critical importance of timely diagnosis for optimal patient outcomes.

## Introduction

Pyoderma gangrenosum (PG) is an uncommon, intense inflammatory neutrophilic dermatosis presenting as painful, recurrent, non-healing ulcers. These ulcers frequently develop in the context of inflammatory triggers [1, 2]. Based on its phenotypic presentation, PG can be classified into several categories: ulcerative, bullous, pustular, vegetative/granulomatous, and peristomal [2]. PG is frequently misinterpreted as recurrent necrotizing soft tissue infections or vasculitis manifestations, which poses challenges in its accurate diagnosis, often leading to a delay in appropriate therapeutic interventions. Notably, even trivial traumas, such as foreign body penetration, can elicit a marked inflammatory reaction termed pathergy, a pathognomonic sign of PG [3].

PG is a distinctive dermatologic condition characterized by recurrent inflammatory ulcers, often manifesting with violaceous borders and undermined edges. The accompanying pain is unusually out of proportion to the degree of ulceration [3]. The classic ulcerative variant is the most encountered phenotype, demonstrated in approximately 85% of all PG cases, with nearly 57% of these lesions afflicting the lower extremity [4].

The reported incidence of PG stands at approximately 0.63 cases per 100,000 individuals and tends to occur at a median age of onset of 60 years, with a notable predilection towards females, accounting for 68% of the cases [5, 6]. Diagnosis for PG relies heavily on a robust clinical suspicion, corroborated by histopathological findings, and necessitates the exclusion of potential secondary etiologies, including infectious processes, vasculitis, and hematologic malignancies [6]. 

## Case presentation

A 40-year-old male, employed as a mechanic for a decade, with a medical history significant for well-managed asthma and hypertension, presented to the emergency department. His chief complaints were an acute exacerbation of a chronic swelling accompanied by a dull-aching, persistent pain in his left index finger, persisting for approximately 10-14 days. Alarmingly, he noted the onset of a blackish discoloration over the affected area two days before seeking medical care. Importantly, he recalled a work-related trauma to the same finger approximately six months prior, where he sustained an inadvertent puncture wound from a slender metallic tube, resulting in an unnoticed foreign body penetration. Post-injury, he sought care at a rural health facility for minor bleeding and swelling, where conservative management was advised, including the application of cold compresses. Although there was a partial resolution of the swelling, it never completely resolved.

Approximately three months following the initial injury, the patient experienced an exacerbation of swelling and intense pain at the injury site, prompting another visit to the emergency department. An X-ray of the left hand was conducted during this visit, revealing the presence of a retained metallic foreign body. The surgery team was promptly consulted, leading to the surgical extraction of the foreign object. While the procedure initially yielded symptomatic relief, the patient's symptoms recurred within weeks, severely impeding his functional capacity at work. Despite repeated outpatient consultations, a myriad of oral and intravenous antibiotic regimens targeting suspected recurrent cellulitis, and several sessions of irrigation and debridement, his persistent symptoms remained notably disproportionate to the observed extent of cellulitis.

During the current hospitalization, vital signs were within normal limits. On physical examination, there was marked erythema and pronounced swelling enveloping the entire left index finger. Additionally, there was a violaceous to black discoloration over the distal phalanx, spanning from the dorsal to the ventral surface, accompanied by erythematous margins. Both active and passive movements of the affected finger were restricted, and palpation elicited significant tenderness.

During the initial diagnostic workup, the patient exhibited mild leukocytosis, with a predominance of neutrophils, and a slight elevation in inflammatory markers (Table 1). The consulting hand surgeon, familiar with the patient from prior inpatient visits, remarked that purulence was never observed during previous interventions. This observation bolstered the likelihood that infection was not a primary factor in the patient's recurrent symptoms. Given the clinical suspicion of pyoderma gangrenosum (PG), the patient was initiated on a three-day regimen of high-dose intravenous methylprednisolone, to be followed by an oral prednisone taper. An ensuing MR angiogram with contrast of the left hand showed no notable vessel stenosis or occlusion. An excisional biopsy of the affected finger revealed subcutaneous tissue showing signs of organization, fibrosis, and granulation tissue interspersed with inflammation. Pertinently, our patient did not present with any underlying chronic autoimmune disorders, nor did he identify with chronic medical conditions, such as diabetes, that might impede wound healing. However, he did exhibit signs of pathergy, likely stemming from his antecedent trauma and the subsequent debridements undertaken for presumed recurrent cellulitis.

**Table 1:** Workup showing leukocytosis and elevated inflammatory markers.

Inflammatory Markers		
C-Reactive Protein (CRP)	1.22	mg/dl
Erythrocyte Sedimentation Rate (ESR)	12	mm/1stHr.
Procalcitonin (PCT)	0.04	ng/ml
White Cell Count (WBC)	16380	cells/mm

Despite the steroid treatment, the patient reported only modest pain relief, although there was a discernible reduction in swelling as evidenced by daily physical examinations. Nonetheless, he continued to necessitate high doses of both opioid and non-opioid intravenous analgesics to manage his intense, unyielding pain. 

**Figure 1:**
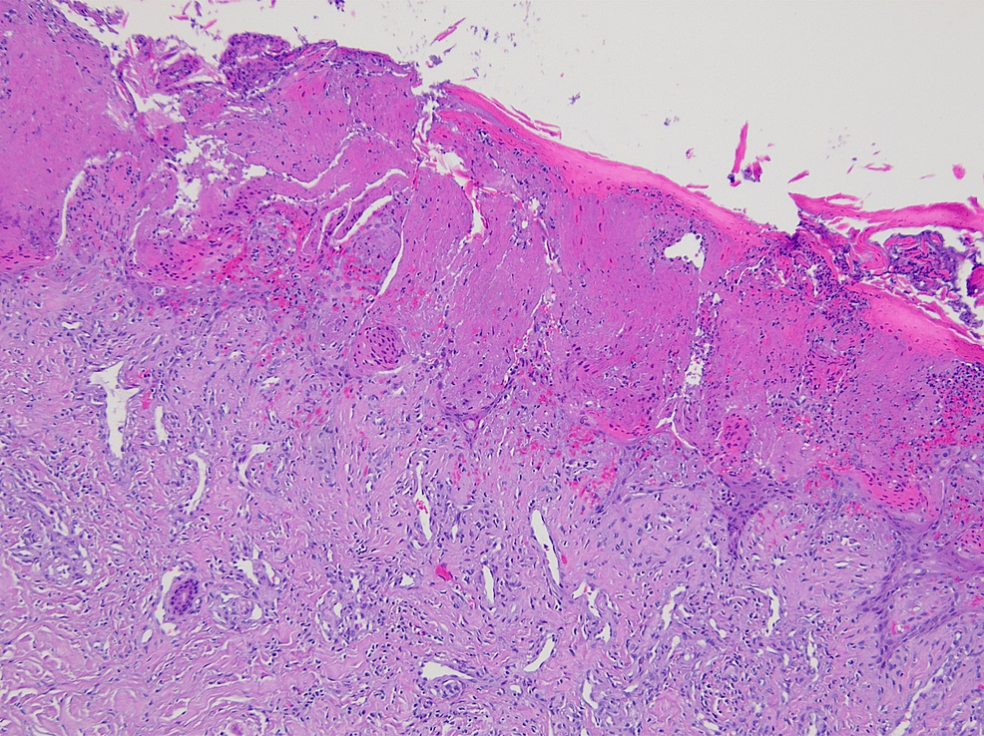
H&E stained image of the epidermis with superficial epidermal necrosis.

**Figure 2:**
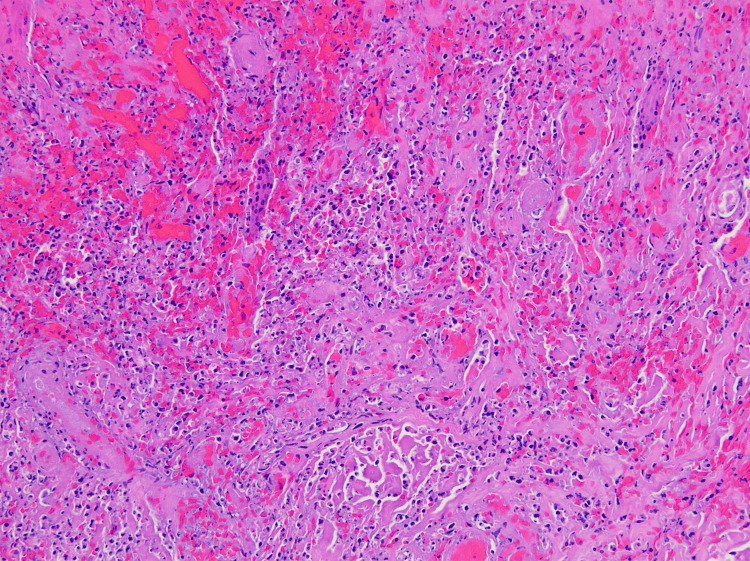
H&E stained image of the dermis showing mixed acute and chronic inflammation with necrosis and hemorrhage at the ulcer's edge.

In consultation with the patient and the hand surgeon, it was determined that amputation of the left index finger was necessary due to the patient's persistent and debilitating pain. The procedure was executed successfully. A peri-procedural biopsy of the removed tissue highlighted superficial epidermal necrosis with extensive hemorrhagic necrosis in the soft tissues and epidermis, accompanied by inflammation and granulation tissue (Figure 1 and Figure 2). These findings were congruent with the clinical diagnosis of pyoderma gangrenosum.

## Discussion

Louis Brocq, a French physician, was the first to identify the characteristic lesions of PG in patients, characterized by rapidly progressing, well-defined necrotic ulcerations with purulent centers. Nevertheless, it was Brunsting et al. who coined the term "Pyoderma Gangrenosum" in 1930 [7].

Historically, PG was believed to have an infectious etiology. Over time, however, it was recognized that the condition results from sterile skin inflammation [8]. The precise pathogenesis remains poorly defined. However, it has been hypothesized to be an interplay of various factors including up-regulation of auto-inflammatory response, stimulation of adaptive immunity by T-cell auto-reactivity, and genetic predisposition [8]. An observed association exists between PG and autoimmune conditions such as inflammatory bowel disease (IBD), arthritis, systemic lupus erythematosus (SLE), monoclonal gammopathy, sarcoidosis, and certain hematologic malignancies. This relationship is believed to be linked to the effects on T-cells and interleukins, offering potential avenues for future therapeutic exploration [8]. Other subtypes of PG include the bullous form, presenting with painful vesicles and bullae; the pustular type, characterized by painful pustules; the granulomatous superficial type, distinguished by verrucous and ulcerative lesions; the peristomal variant, typically occurring around stomas; and the rare malignant pyoderma, notable for severe ulceration without the classical border [9].

PG is commonly misdiagnosed as uncomplicated non-healing ulcers. Consequently, patients might undergo debridement, potentially inducing pathergy reactions that can exacerbate the condition. In the presented case, a metallic wire puncture to the patient's left index finger, followed by foreign body removal, might have initiated the pathergy. Subsequent surgical interventions likely intensified the clinical manifestations due to ensuing pathergy reactions. A skin biopsy is frequently conducted to corroborate the PG diagnosis. Its primary utility lies in excluding other etiologies of cutaneous ulcers, especially infectious origins. Biopsy should target the active edge of the ulcer and subcutaneous tissues deep within the ulcer. It is imperative to inform patients that such procedures often result in an expanded ulceration and a potential pathergy reaction due to the trauma [10].

Cutaneous neutrophilic abscesses are commonly observed in biopsies of early-stage PG lesions. As lesions progress to their later stages, there's evident necrosis and ulceration at the epidermal level, accompanied by edematous changes in the superficial dermis. Additionally, there's a pronounced combined cellular infiltration within the dermis, occasionally extending into the underlying panniculus adiposus. In our case, the pathological findings aligned with the clinical suspicion of PG, characterized by extensive hemorrhagic necrosis of the soft tissue and skin, admixed with inflammation and granulation tissue.

Establishing a diagnosis of PG is intricate due to its varied clinical presentations and the lack of definitive diagnostic assays. Primarily, diagnosis rests on clinical evaluations, ruling out other potential causes, and histopathological evaluation of skin biopsies, which demonstrate a marked neutrophilic infiltrate in the absence of infectious evidence. Notably, patients often exhibit raised erythrocyte sedimentation rates coupled with neutrophilic leukocytosis.

In 2018, Maverakis et al. introduced an updated diagnostic framework for ulcerative PG, formulated through a Delphi consensus of international experts [8]. This diagnostic model comprises one major criterion and eight minor criteria. For a definitive diagnosis, both the major criterion and a minimum of four minor criteria must be met [6]. The Delphi exercise identified one major criterion - a biopsy from the edge of the ulcer revealing a neutrophilic infiltrate - and eight minor criteria: (1) exclusion of infection; (2) pathergy; (3) history of inflammatory bowel disease or inflammatory arthritis; (4) history of papule, pustule, or vesicle ulcerating within four days of appearing; (5) peripheral erythema, undermining border, and tenderness at ulceration site; (6) multiple ulcerations, at least one on an anterior lower leg; (7) cribriform or "wrinkled paper" scar(s) at healed ulcer sites; and (8) decreased ulcer size within one month of initiating immunosuppressive medication(s).

The PARACELSUS score [11] serves as a sensitive diagnostic instrument for PG. The diagnosis of Pyoderma Gangrenosum involves three major criteria: rapidly Progressing disease, Assessment of relevant differential diagnoses, Reddish-violaceous wound border, and seven minor criteria (Amelioration by immunosuppression, Characteristic irregular shape, Extreme pain, Localization at the site of trauma, Suppurative inflammation, Undermined border, and Systemic disease associated). These criteria are encapsulated in the mnemonic 'PARACELSUS.' A cumulative score of 10 or above strongly suggests a diagnosis of PG.

In patients suspected of having PG, a comprehensive evaluation, including a biopsy, is essential to rule out conditions that mimic PG [12]. The differential diagnosis includes antiphospholipid antibody syndrome, venous stasis ulcers, vasculitis, cutaneous infections, factitial ulcers, vascular occlusion disorders, and malignancies [8].

To our current understanding, no definitive guidelines exist for the management of PG. The cornerstone of treatment primarily revolves around pain management and immunosuppression, using steroids. It is also essential to concurrently address disease activity suppression, manage secondary infections, control inflammation, and treat the underlying disease.

For localized and minor lesions, approximately smaller than 3 cm in diameter, topical treatments like corticosteroids, calcineurin inhibitors (such as tacrolimus or pimecrolimus), or specific dressings might suffice [13]. For more extensive or multiple lesions, systemic interventions become essential. Commonly administered systemic treatments include oral corticosteroids like methylprednisolone or prednisone, cyclosporine, and biologic agents such as infliximab and adalimumab, often spanning several months [13]. In cases marked by pronounced tissue damage, surgical measures, ranging from debridement to skin grafting, might be warranted.

In the presented case, upon suspicion of PG, intravenous steroids were administered considering the lesion's severity. While the patient demonstrated clinical improvement, he opted for a left index finger amputation due to persistent, severe pain. The surgical procedure was executed successfully, with no intraoperative purulence detected and clean surgical margins observed.

PG is characterized by a relapsing and remitting course, with phases of active ulceration and subsequent healing. While the recurrence rates vary, studies indicate that approximately half of the patients might experience repeated episodes [8].

Pyoderma Gangrenosum is often a diagnosis of exclusion and can be mistaken for other conditions, so reliable, targeted objective markers would greatly aid in differentiating PG from similar dermatological disorders. Furthermore, the histopathological findings in PG can vary, making it nonspecific and dependent on the stage of the lesion [14]. Thus, the establishment of standardized, specific histologic criteria for PG could lead to an earlier and more accurate diagnosis, which is crucial for the initiation of appropriate treatment. This is particularly important given that misdiagnosis and delayed treatment can lead to severe consequences, as seen in this case. Therefore, the development of targeted objective markers and standardized specific histologic criteria for PG is of paramount importance.

## Conclusions

Persistent tender lesions, either unresponsive to antibiotics or associated with negative cultures, especially when paired with other indicative systemic conditions, should prompt consideration of alternative diagnoses such as PG. When PG is a strong clinical suspicion, clinicians must prioritize obtaining a biopsy for a definitive diagnosis. Such proactive measures are crucial in circumventing unnecessary interventions and halting potential exacerbations due to pathergy.

In our presented case, the lack of coexisting autoimmune or chronic inflammatory conditions adds to the intricacy of diagnosing PG. The patient's history of foreign body penetration led to multiple emergency department consultations, each predicated on the assumption of recurrent cellulitis. His clinical journey was further complicated by repeated debridements, possibly intensifying the pathergy. Tragically, this prolonged misdiagnosis led to the patient's decision to amputate the affected finger owing to intolerable pain. We therefore emphatically stress the importance of early recognition of the salient features of PG to avert therapeutic delays.

It is paramount for clinicians to understand potential manifestations of PG, even in the absence of associated systemic conditions. Clinicians must remain vigilant regarding the diagnostic challenges associated with PG, ensuring patients receive the best opportunity for timely and effective treatment outcomes.
